# Classification of Coffee Beans by GC-C-IRMS, GC-MS, and ^1^H-NMR

**DOI:** 10.1155/2016/8564584

**Published:** 2016-07-18

**Authors:** Victoria Andrea Arana, Jessica Medina, Pierre Esseiva, Diego Pazos, Julien Wist

**Affiliations:** ^1^Grupo de Investigación Ciencias, Educación y Tecnología (CETIC), Programa de Química, Facultad de Ciencias Básicas, Universidad del Atlántico, km 7 Antigua Vía Puerto Colombia, Barranquilla, Atlántico, Colombia; ^2^Chemistry Department, Universidad del Valle, A.A. 25360, Cali, Colombia; ^3^Institut de Police Scientifique, École des Sciences Criminelles, Université de Lausanne, 1015 Lausanne, Switzerland

## Abstract

In a previous work using ^1^H-NMR we reported encouraging steps towards the construction of a robust expert system for the discrimination of coffees from Colombia versus nearby countries (Brazil and Peru), to assist the recent protected geographical indication granted to Colombian coffee in 2007. This system relies on fingerprints acquired on a 400 MHz magnet and is thus well suited for small scale random screening of samples obtained at resellers or coffee shops. However, this approach cannot easily be implemented at harbour's installations, due to the elevated operational costs of cryogenic magnets. This limitation implies shipping the samples to the NMR laboratory, making the overall approach slower and thereby more expensive and less attractive for large scale screening at harbours. In this work, we report on our attempt to obtain comparable classification results using alternative techniques that have been reported promising as an alternative to NMR: GC-MS and GC-C-IRMS. Although statistically significant information could be obtained by all three methods, the results show that the quality of the classifiers depends mainly on the number of variables included in the analysis; hence NMR provides an advantage since more molecules are detected to obtain a model with better predictions.

## 1. Introduction

*Colombian coffee* is a protected geographical indication (PGI), a recognition for its high quality, and the result of decades of efforts and strategies to federate more than half a million coffee growers. Moreover, the consumption of high profile coffees from specific origins is constantly increasing, meaning that the* 100% Colombian coffee* label represents an economical* plus-value* for local growers. In addition, Colombia imports coffee from neighboring countries to supply its internal market. This context calls for methods able to ensure the quality and origin of the coffee beans. Ideally, they should be sufficiently robust and cost-effective to be implemented at harbour installations for screening of material immediately after arrival or just before being shipped.

Several efforts have been directed to this aim; the stable isotopes composition, such as *δ* ^2^H, *δ* ^13^C, *δ* ^15^N, and *δ* ^18^O, determined by Isotope Ratio Mass Spectrometry (IRMS) has been used as markers for environmental conditions and agricultural practices, as well as for the identification of the origin of coffee [1, 2]. Using the same technique, Rodrigues et al. [3] determined that the differences in geographic location are due mainly to altitude and precipitation. Near-Infrared Spectroscopy (NIRS), Mass Spectrometry (MS), and Nuclear Magnetic Resonance (NMR) have initially been applied to determine the composition of mixtures of* Coffea arabica* L. and* Coffea canephora* var.* robusta* [4–13], before targeting the determination of the origin of coffee beans. Hyphenated separation methods coupled to Mass Spectrometry, such as HS-SPME-GC-TOF-MS for the detection of volatile and semivolatile compounds and LC-MS and GC-FID for the quantification of amino acids and carbohydrates, could also distinguish coffees from different origin [14, 15].

A very nice review by Kelly et al. [16] lists the different techniques that have been successfully applied to the determination of origin of food products, while an up-to-date list of articles that report on the determination of origin of coffee can be found in Table 1. At first glance, it can be appreciated that a wide range of methods have been evaluated and second that only a small fraction of contributions are based on NMR. More interestingly, looking at the sampling schemes reported, in particular the choice of countries per continents, indicates that these results should be considered with care and that further evaluations are required to evidence the potential of such methods for precise localization of samples, for example, to distinguish between neighboring countries. In addition, the sources of variance that may influence the profiles in one country, altitude, precipitations, postharvest processing, and so forth, are hardly accounted for in such experimental design. Last but not least, another important issue is uncovered from Table 1: most contributions report on a single technique making comparison between them very impractical.

In a previous work [17] we have shown that ^1^H-NMR led to accurate discrimination of roasted Colombian beans when using samples collected during several years and from all over Colombia forming, in a good approximation, a representative set of Colombian coffees. In addition, a large set of samples from the same period of time and collected from all over the world formed the best possible approximation to a representative set of non-Colombian samples. In this contribution a subset of these collections was carefully chosen to retain most of the sources of variance and was analyzed using two additional methods as an attempt to obtain a fair comparison of their abilities to distinguish coffees from Colombia, Peru, and Brazil. In Section 2 we will describe the sample set, the preparation protocols, and the chemometrics methods. In Section 3 we present our results. Finally, conclusions are provided in Section 4.

## 2. Materials and Methods

### 2.1. Samples Collection and Preparation

A total of 34 samples of roasted coffees (*Coffea arabica* L.) were collected over a two-year period, 2012 to 2013, from 3 different countries of South America. The samples were provided by Almacafé S.A. (Colombia) and distributed as follows: 15 samples from Colombia, 11 from Brazil, and 8 from Peru. Colombian coffee samples were distributed as follows: 2 samples came from coffee farms located in the Department of Tolima, 4 samples came from Huila, and 9 samples came from Nariño. Each delivered parcel contained samples from different origins according to harvests.

It is important to highlight that sampling took place at regional collection centers, where coffee grains from the region are checked for quality and stored together. This means that the origin of the beans is duly controlled by collecting authorities, in a manner very close to real implementation of the tool. This also means that no fine grain geographic data such as GPS coordinates are available for this study, which is not relevant to the specific purpose of comparing different analytical techniques.

Batches were analyzed in random order. The sample preparation process for GC-C-IRMS experiments was the one proposed by Weckerle et al. [19] and starts with a liquid-liquid extraction. First, 80 mg of coffee powder was extracted in 1 mL of boiling water during 10 min agitation by vortex. The filtered solution was subjected to liquid-liquid extraction with 1 mL of chloroform during 10 min vortex. The organic phase was dried over anhydrous Na_2_SO_4_ and filtered. Subsequently, 180 *μ*L of extract was transferred to a vial and 20 *μ*L of tetradecanoic acid methyl ester (40 *μ*g/mL) was added as internal standard. For GC-MS samples, 200 mg of finely ground coffee was extracted at room temperature in 1 mL of dichloromethane. After two-minute agitation with vortex, the samples were filtered and transferred to a vial with 0.2 mg/mL of 1-decanol as internal standard. For ^1^H-NMR, 200 mg of finely ground coffee powder was extracted at room temperature in 1 mL HPLC grade methanol. After two-minute agitation with vortex, the samples were centrifuged for 10 min at 17°C and 450 *μ*L of the extract was transferred to the NMR tube. Last, 90 *μ*L of deuterated methanol with TMS was added.

### 2.2. Analytical Techniques

#### 2.2.1. GC-C-IRMS

The *δ* ^13^C values of caffeine were determined with a Delta V Advantage Isotope Ratio Mass Spectrometer (IRMS) system (Thermo Fisher Scientific, Bremen, Germany) coupled to a Trace GC Ultra Gas Chromatograph via a GC-C/TC III interface operating in the “Combustion” (C) mode and equipped with a TriPlus*™* autosampler. DB-17MS GC column was used for the separation with the following operating conditions: injection temperature, 280°C; oven temperature, 70°C for 2 min, ramp at 15°C/min to reach 160°C, ramp at 10°C/min up to 280°C, and constant temperature for 2 min. The total run duration was of 32 minutes. A constant flow (1.6 mL/min) of helium gas was injected as carrier gas and 1 *μ*L of sample solution was injected in splitless mode. The detection of ions at 44, 45, and 46* m/z* is carried out by an impact source with a 3 kV acceleration voltage, a magnetic field, and a Faraday collector for the measurement of each mass. The temperatures inside the combustion and reduction ovens were of 940°C and 600°C, respectively. Six pulses of reference gas, CO_2_, of 20 s each were introduced during the chromatographic separation. Complete oxidation of the combustion chamber was performed after each batch of 20 samples. Randomly chosen duplicates were intertwined between runs to check for experimental error. Acquisition and evaluation of GC-C-IRMS data were performed with software ISODAT 2.5 (Thermo Fisher Scientific, Bremen, Germany). After the complete conversion of the C atoms into CO_2_ at 1000°C and separation of the water vapor, the carbon isotope ratio was determined by measuring the 3 masses, 44, 45, and 46. The relative *δ* ^13^C values were calculated with respect to the reference gas (CO_2_), where the symbol *δ* is the standard notation to express the carbon isotope ratio. The latter is defined as parts-per-thousand deviation from the isotopic composition of Vienna Pee Dee Belemnite (VPDB) and is calculated according to [31]:

(1) $$\begin{array}{c} \delta^{13}C_{mu}\left(\;\text{‰}\;\right)=\left[\;\left(\;\frac{R_{mu}}{R_{st}}\;\right)-1\;\right]*10^{3}. \end{array}$$

*R*_mu_ and *R*_st_ correspond to the sample and to the standard ^13^C/^12^C isotope ratio, respectively. The *δ* ^13^C CO_2_ isotope ratio was determined using the international standard, tetradecanoic acid methyl ester, whose isotope ratio is *δ* ^13^C = −29.98. Each sample was analyzed in duplicate.

#### 2.2.2. GC-MS

The GC-MS analysis was carried out with a gas chromatograph 6890N (Agilent Technologies, Palo Alto, CA), equipped with the same column, as mentioned above, a DB-17MS GC column (length: 30 m, inner diameter: 0.25 mm, and film thickness: 0.25 mm), coupled to an inert mass selective detector model 5975 and an autosampler HP 7673 (Agilent Technologies, Palo Alto, CA). Randomly chosen duplicates were included in each batch to check for experimental error. The syringe was rinsed three times with dichloromethane and then with the sample solution prior to and after injection. 1 *μ*L of sample solution was injected in split mode (10 : 1). Operating conditions were as follows: injection temperature, 280°C; oven temperature kept at 50°C for 2 min, ramp at 10°C/min to reach 220°C, ramp at 5°C/min up to 300°C, and constant temperature for 5 min, for a total of 40 min. Helium gas was used as carrier in constant flow mode (1.0 mL/min) with a linear velocity of 36.0 cm/s. The detection was set in electron impact mode (70 eV) in order to observe masses from 50 to 500* m/z*. The temperatures inside the transfer line and the ion source were set at 250 and 230°C. The identification of the compounds was carried out by comparison with spectra of pure reference compounds and with the Wiley library [32]. Identified compounds were quantified by integration of their signals. Each integral was normalized with respect to the sum of the integrals present in each trace, thus computing relative intensities for each compound [6] (see Supplementary Material, File 1, available online at http://dx.doi.org/10.1155/2016/8564584).

#### 2.2.3. ^1^H-NMR

All NMR experiments were performed on a 400 MHz Bruker spectrometer using a BBO probe head with triple-axis gradients and automated tuning and matching accessories. Accurate control of the sample temperature was achieved using a BVT-1000 and BCU-1 units. Samples were measured at 300 K in fully automatic mode, with the help of a Sample Express changer accessory. Randomly chosen duplicates were analyzed with each batch to check against experimental error.

The acquisition of the spectra was achieved as described elsewhere [17]. Three experiments were run sequentially. First, a simple one-pulse experiment (zg30) with a 1 s relaxation delay, a short excitation pulse (0.1 *μ*s), and a 4 s acquisition time was used to estimate the frequencies of methanol and to build a cosine modulated shape pulse of 1 s (25 Hz) with 50.000 complex data points for band-selective saturation.

The second experiment (zgps) was run using a 6 s band-selective saturation achieved by the just mentioned pulse. The resulting spectra were used to refine the frequencies measured for the two signals of methanol.

The final experiment (noesygpps) was recorded with a receiver gain of 90.5, a mixing time of 10 ms, 4 dummy scans, and 64 FIDs. The resulting FIDs were apodized using a 0.3 Hz exponential function prior to Fourier transformation and only zero order phase correction was allowed.

### 2.3. Chemometrics

PCA was used to check data quality and identify possible outliers or detect unexpected aggregation. Box-plots and ANalysis Of VAriances (ANOVA) were used for an initial assessment of the information provided by the techniques that is relevant to discrimination by country of origin. Here we were looking for variables that are distributed significantly different on the subset of Colombian samples relative to the rest of the samples. Then, multivariate analysis was performed by means of Partial Least Squares-Discriminant Analysis (PLS-DA) models for classification according to country of origin (Colombia versus other countries). These models were validated using the 7-fold Cross-Validation (CV) method. Average *Q* ^2^ values (as defined by Szymańska and collaborators [33]) over the 7 models were used as indicators of quality and for the determination of the best number of components (at the turning point of *Q* ^2^ versus number of components curve). Variable-Importance-in-Projection (VIP) estimators [34] were then computed for each predictor and provided us with an estimation of which and how many predictors are relevant to the classification.

Because PLS-DA is a supervised method that learns from data with known labels, the quality of the model may vary from one CV sampling to another. Thus, simply comparing *Q* ^2^ estimators of two models obtained with different parameters, for instance, with different numbers of predictors, is meaningful only as long as the width of the distribution of *Q* ^2^ values arising from resampling is much lower than the effect of interest. However the magnitudes of these effects are not known* a priori*. A workaround consists in comparing the manners in which *Q* ^2^ values vary among a large number of models, 100 in our case, computed for each condition to be compared, that is using either all the available predictors or a subset of them.

Analyses were run using SIMCA (http://umetrics.com/products/simca) and the caret package [35] of the R statistical software [36].

## 3. Results

### 3.1. GC-C-IRMS

**Figure 1:**
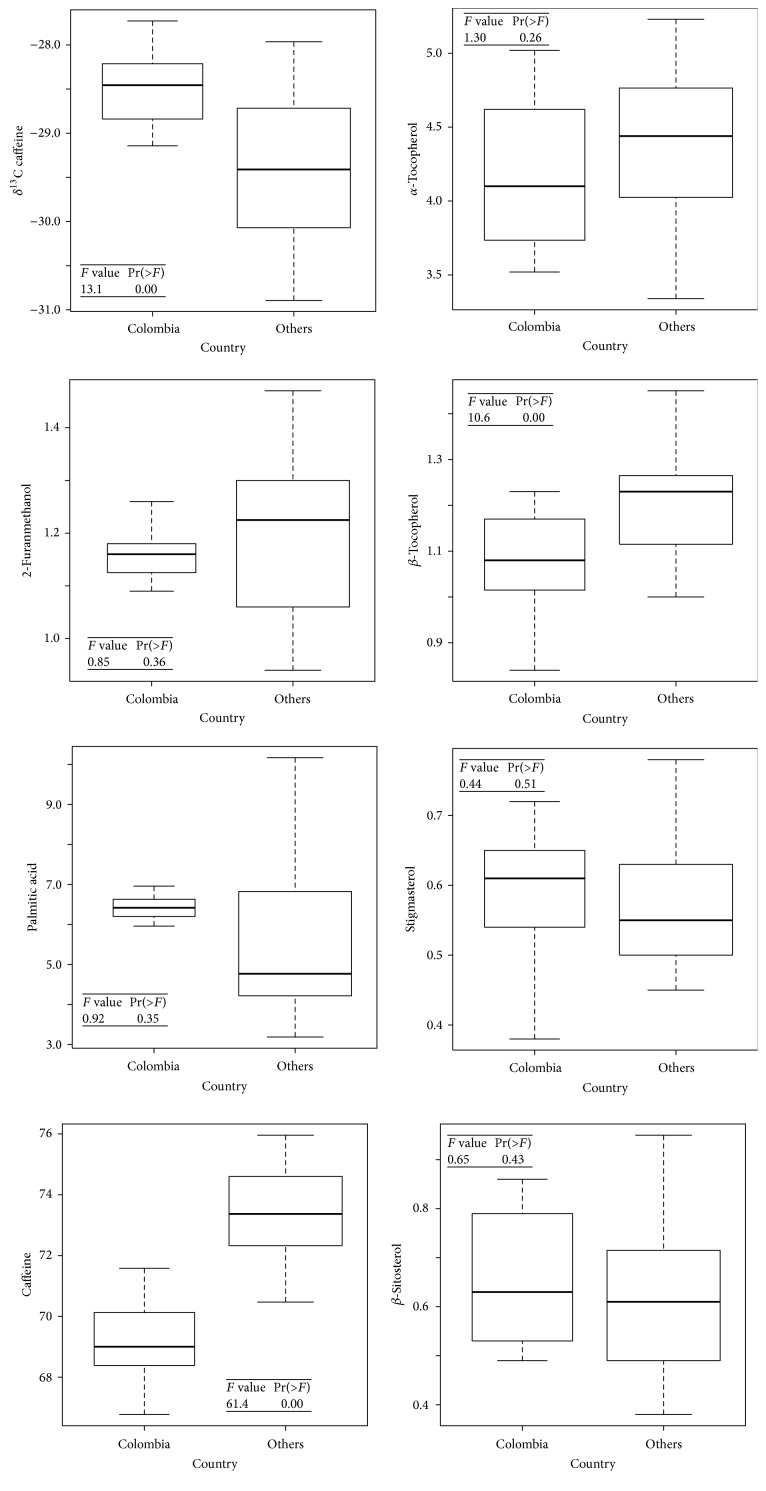
Box-plots for caffeine *δ* ^13^C ratios obtained by GC-C-IRMS and for the 7 compounds quantified by GC-MS. The *F* values and corresponding *p* values were computed by ANOVA for each predictor. Although several predictors were found to be significantly different for both groups, the overlap observed between the distributions already suggested that the accuracy of the classifier would depend on the number of predictors included in the analysis.

Figure 1 (upper left) shows the box-plots for the *δ* ^13^C ratio of caffeine in coffee samples from Colombia versus other countries, along with the corresponding *F* values given by ANOVA. It is found that the distributions are significantly different, with *F* = 13.1. Yet, the overlap observed in the box-plots immediately suggests that a classifier based solely on this predictor would incur significant error. These findings agree with results reported by Weckerle and collaborators [19]. Indeed the authors found, using fewer samples from each country, significant overlap of the *δ* ^13^C isotope values of Brazil, Colombia, and Costa Rica and observed an improvement in their classifications when ratios for other elements, in particular *δ* ^18^O, were included.

### 3.2. GC-MS

A Principal Component Analysis performed with the relative abundance of 7 compounds (2-furanmethanol, palmitic acid (C16:0), caffeine, *α*-tocopherol, *β*-tocopherol, stigmasterol, and *β*-sitosterol) extracted from the chromatograms allowed spotting one outlier that was removed from further analysis.

The remaining 7 box-plots of Figure 1 display the distribution in the concentrations of the 7 compounds detected by GC-MS (2-furanmethanol, palmitic acid (C16:0), caffeine, *α*-tocopherol, *β*-tocopherol, stigmasterol, and *β*-sitosterol) when comparing Colombian samples with other samples. Two compounds presented significant differences according to ANOVA (caffeine: *F* = 61.4 and *β*-tocopherol: *F* = 10.1 (degrees of freedom (dof) = 1 and 32)). Furthermore, though caffeine distributions here present a larger gap than in the case of GC-C-IRMS, they still overlap by a full quartile, and the overlap is even larger for *β*-tocopherol. No single GC-MS variable then provides a good enough classifier, but multivariate analysis may still solve the task by combining multiple predictors.

**Figure 2:**
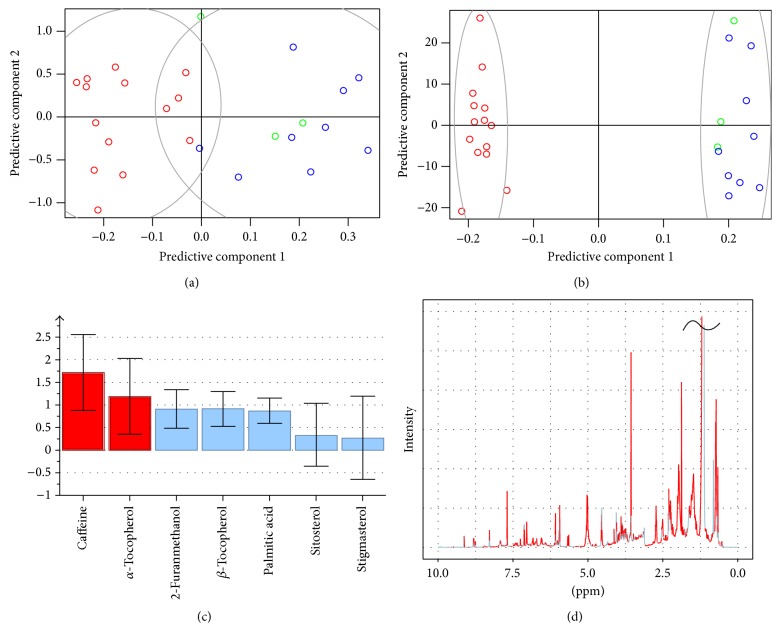
Score plots of PLS-DA for classification by country of origin (Colombia versus Others) performed with GC-MS (2 LVs, (a)) and ^1^H-NMR (8 LVs, (b)). Red, green, and blue open circles are for Colombia, Peru, and Brazil, respectively. (c) VIP plots obtained for GC-MS model. (d) NMR spectra colored according to the results of VIP analysis. The red color (VIP > 1) means that the variable contributes to prediction, while the light blue color (VIP < 1) is for predictors that do not contribute. VIP scores obtained from NMR show that most of the spectra (peaks) are contributing to the classification, highlighting that a large number of observable compounds are important for the determination of origin of coffee beans. A total of 662 predictors were found with VIP > 1.

 Figures 2(a) and 2(c) summarize the results of PLS-DA on the set of GC-MS predictors. Models were built with 2 latent vectors (LVs), which allowed for the best results while avoiding overfitting. The scores plot of a randomly selected model (Figure 2(a)) shows how this method achieves some success in discriminating Colombian coffee samples (*Q* ^2^ = 0.702) but still presents overlap between the classes. On the other hand, VIP analysis singles the predictors that were most relevant to the classification, which again turn to be caffeine and *α*-tocopherol.

In the end, we conclude that GC-MS provides two useful predictors of coffee origin but that they are still insufficient to achieve a robust classification.

### 3.3. ^1^H-NMR

Figures 2(b) and 2(d) summarize the results of PLS-DA on the NMR dataset. Models were built with 8 LVs. Simple visual inspection of the results reveals a much better classification than the one obtained by GC-MS. This is confirmed by the average classification quality factors obtained, which were *R* ^2^ = 0.69 compared to 0.66 from GC-MS and *Q* ^2^ = 0.85 compared to 0.702.

Once more, VIP plots permit identifying some of the relevant variables, in this case signals that can then be attributed to relevant molecules. The results of this analysis are presented in Figure 2(d). A total of 662 chemical shifts located all across the spectra range were found to be of significance for the classification.

## 4. Discussion

These results suggest that ^1^H-NMR's success is not linked to some key variables/compounds that it manages to detect (targeted approach), but to the combined amount of information observed simultaneously (nontargeted approach). Furthermore, this agrees with the overall progression observed for the techniques evaluated here: only one GC-C-IRMS variable is available, which though significatively discriminant, as revealed by ANOVA, is clearly not enough to yield a good classificator. Then, GC-MS targeted 7 compounds, 2 of which turned to be significant for origin discrimination. These predictors managed to allow for a PLS-DA classification that though still unsuitable attained better class separation than the sole GC-C-IRMS variable. Last, ^1^H-NMR achieved the best results, not through some predictor with extraordinary discriminant power, but by the conjunction of many significant variables. In the hypothetical case where isotope ratios could be measured simultaneously for several elements and for a large set of compounds, IRMS would certainly provide more robust classifiers, as would GC-MS if more compounds could be quantified simultaneously.

**Figure 3:**
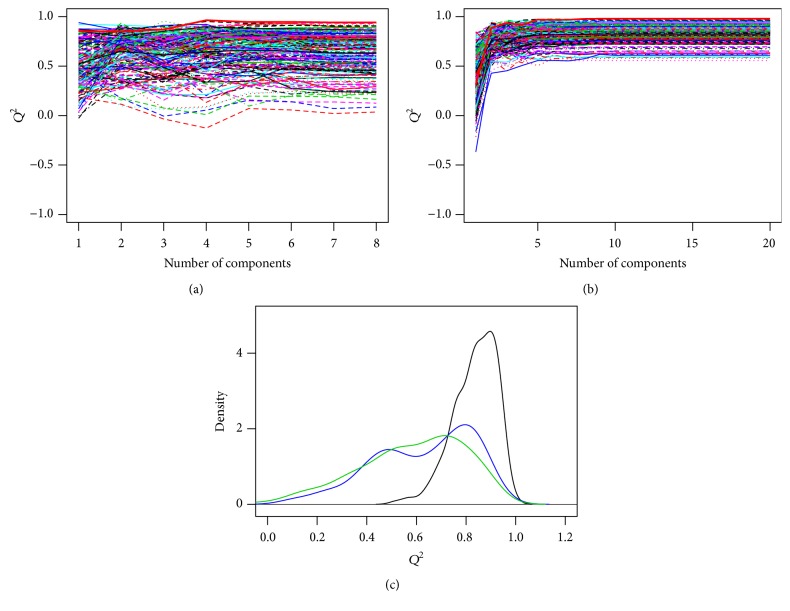
(a) and (b) show the behavior of *Q* ^2^ as a function of the number of latent vectors (LVs) for 100 models sampled randomly for GC-MS (7 predictors, (c)) and NMR (1610 predictors, (b)). The thick red curve represents the average of all models and its turning point is used to determine the best number of components for which the distributions are shown on (c). The green curve represents the distribution of *Q* ^2^ for 2 LVs (GC-MS). The blue and black curves are for NMR using 2 and 8 LVs, respectively, and using either all the predictors (black) or only the best 8 predictors (blue), selected according to their VIP.

Figure 3 presents the results of the analysis of the “sensitivity” of the multivariate models towards resampling. For this purpose, 100 models were built for GC-MS using 7 predictors and for NMR using either all the predictors (1610) or only the 8 predictors with the highest VIP. Once more, the number of predictors turned to be the key factor: *Q* ^2^ values of the full NMR model were much less sensitive to resampling than those of the models with fewer predictors, as revealed by the width of the corresponding distributions.

These results fuel the hypothesis that origin determination cannot be reduced to a handful of compounds, regional markers. Instead, geographical origin manifests itself in the form of subtle modulations of the concentration of a large variety of compounds. After all, coffee beans from the same variety and species, harvested and processed in a similar fashion, cannot be expected to differ drastically in their chemical composition.

This also means that any attempts to combine data from all three techniques will not lead to more accurate classifications due to the unbalanced number of variables. Indeed, a bunch of isotope ratios and GC-MS intensities will be marginalized by the overwhelming 662 predictors obtained by NMR, obscuring any benefit of adding potentially relevant predictors.

## 5. Conclusions

The results are reported for the classification of roasted coffees from nearby countries, Colombia, versus Peru and Brazil (Others), using three different analytical techniques that have been previously shown to be promising for such task. Unlike the reports the authors are aware of, where classification is achieved between coffee samples of very distinct geographic areas, the present work focuses on high profile coffees produced in nearby countries, hence very similar samples, in order to explore the limits of the abovementioned methods. Commonly accepted statistical analyses were performed and standard procedures were used to acquire the data in an attempt to obtain a fair comparison of what could be achieved in a laboratory in charge of quality control and at a reasonable cost.

The results reported in this work show that when trying to determine the origin of coffees the number of predictors that can be observed is pivotal; hence NMR fingerprinting that allows the simultaneous observation of a very large number of compounds is found to perform best. This conclusion is supported not only by the accuracy of the predictions that is higher than for the other two methods, but also by the VIP analysis showing that many regions of the spectra are relevant to the classification. In other words, the origin of coffee is encoded by subtle modulations of the concentration of many different compounds.

This puts the cost-effectiveness of the techniques studied under a different light: NMR demands complex logistics associated with cryogenic magnets, an issue that was the initial motivation of the present work; however, the result of this investment is a very large amount of information (over a thousand variables), most of which turned out to be not only significant but also essential for reliably distinguishing Colombian coffee from potential frauds. On the other hand, a technique such as GC-C-IRMS, while less demanding in terms of equipment, implies a time-expensive experimental protocol that in the end leads to a single output variable, far insufficient for the task at hand. In this regard, improved results could be obtained by measuring other isotopic ratios, but that would increase the experimental time and still fall short of the vast amount of information that NMR brings to the table.

These results encourage the exploration of techniques that better balance low cost with high resolution fingerprinting. Cheaper spectroscopic techniques such as IR thus appear as attractive candidates to achieve the ideal of coffee fraud detection at harbour. Further studies by our group exploring this alternative will be published in the near future.

## Supplementary Material

The authors strongly support open data initiatives. Therefore, all the original datasets acquired for this manuscript are made available as supplementary material. It consists of tables, in the case of GC-C-IRMS and GC-MS and of binned spectra in the case of NMR. All data are provided in csv format to make their importation easier. Original raw data may be shared upon request to the authors.

## Figures and Tables

**Table 1:** Scientific articles on determination of origin of coffee.

Analytical technical	Presentation of coffee	Number of samples	Countries	Chemometric tool	Metabolites	Reference
ICP-AES	Roasted	160	Costa Rica (20), Colombia (20), Kenya, Guatemala, Panama, Sulawesi, Ethiopia, Sumatra	PCA, CDA, DFA, ANN	Multielements (19)	[18]
EA-P-IRMS	Green	45	Ethiopia, Malawi, Yemen, Zambia, Kenya, Tanzania, Brazil (5), Colombia (3), Guatemala, Mexico, Costa Rica, India, Sumatra, Jamaica, Hawaii	LDA, CART	Stable isotope ratios of C, H, O	[19]
IRMS	Green	46	Guatemala, Panama, Costa Rica, Brazil, Mexico, Venezuela, Nicaragua, Salvador, Honduras, India, Timor, Papua NG, Sri Lanka, Cameroon, Ethiopia, Uganda, Rwanda, Kenya, Zimbabwe	PCA	Stable isotope ratios of C, N, B	[1]
NIRS	Green	120		PCA	Moisture, fat, protein, sucrose, caffeine, trigonelline, organic acids, chlorogenic acids	[20]
GC-TOF-MS	Roasted	47	Production area: Brazil (11) and Colombia (8). Markets of coffee: Brazil (2), Colombia (12), Costa Rica, Guatemala	PCA	Volatile and semivolatile compounds	[14]
IRMS/EA	Green	68	4 R (Angola), 62 A (Papua NG, Ethiopia, Tanzania, Kenya, Hawaii, Costa Rica, Jamaica, Malawi, Guatemala, Brazil (8), East Timor, Peru (2), Ecuador, Mexico, Salvador, Nicaragua, Zambia, Rwanda, Indonesia)	PCA	Stable isotope ratios of C, N, O and percentage composition of C and N	[3]
HPLC	Green	107	57 A (Guatemala, Cameroon, Congo, Uganda, India, Indonesia, Java, Vietnam), 50 A (Brazil (6), Colombia (1), Costa Rica, Salvador, Guatemala, Honduras, Mexico, Nicaragua, Panama, Venezuela, Cameroon, Congo, Ethiopia, Kenya, Rwanda, Uganda, Zimbabwe, India, Indonesia, Java, Papua NG, Timor, Vietnam)	PCA, LDA, PLS-DA, CART	Chlorogenic acids, amides cinnamoyl, cinnamoyl glycoside, phenolic acids	[21]
LC-MS/ GC-FID	Roasted	21	Asia (4), South America (11), Africa (6)	PCA	Total protein, total carbohydrates, monosaccharides, amines	[15]
IRMS/MC-ICP-SFMS	Green	47	5 different Hawaii coffee-producing regions	CDA	Stable isotope ratios of C, N, S, O, Sr and multielements (30)	[22]
MC-ICP-MS/ IRMS	Green	60	Rwanda, Ethiopia, Tanzania, Kenya, Malawi, Zambia, Zimbabwe, Hawaii, Mexico, Costa Rica, Guatemala, Peru, Salvador, Nicaragua, Brazil, Ecuador, Jamaica, Indonesia, East Timor, Papua NG	PCA	Stable isotope ratios of Sr and O	[23]
ICP-MS/ IRMS	Green	64	Mexico, Guatemala, Honduras, Costa Rica, Salvador, Dom. Rep., Colombia (4), Brazil (6), Uruguay, Ivory Coast, Cameroon, Congo, Ethiopia, India, Indonesia	CDA	Stable isotope ratios of H, C, N, O and multielements (54)	[2]
^1^H-NMR	Roasted	40	Cape Verde, Ethiopia, Kenya, Malawi, Saint Helena, Tanzania, Brazil (5), Colombia (2), Costa Rica, Salvador, Galapagos, Hawaii, Peru (2), Guatemala, Honduras, Jamaica, Nicaragua, India, Sumatra, Nepal, Yemen	OPLS-DA	Chlorogenic acids, trigonelline, lactate, caffeine, fatty acids	[12]
^13^C-NMR	Green	60	Brazil (10), Colombia (10), Guatemala, Tanzania, Indonesia, Vietnam	PCA, OPLS-DA	Sucrose, caffeine, chlorogenic acids, choline, amino acids, organic acids, trigonelline	[13]
ICP-MS/ICP-AES	Green and roasted	42	Kenya, Ethiopia, Uganda, Indonesia, India, East Timor, Australia, Papua NG, Cuba, Dom. Rep., Costa Rica, Peru (1), Guatemala, Colombia (3), Brazil (2)	LDA, PCA	Multielements (59)	[24]
FT-IR	Green	18	4 different Brazil coffee-producing regions	ANN, SIMCA, MLP		[25]
MC-ICP-MS	Green	21	Taiwan, Ethiopia, Kenya, Tanzania, Malawi, Rwanda, Uganda, Brazil, Colombia, Peru, Salvador, Guatemala, Costa Rica, Puerto Rico, Jamaica, East Timor, Indonesia, Papua NG	PCA	Stable isotope ratios of B and Sr and multielements (7)	[26]
^1^H-NMR	Green and roasted	340	Brazil (19), Colombia (70), Ecuador, Peru (16), Hawaii, Costa Rica, Dom. Rep., Mexico, Guatemala, Honduras, Nicaragua, Uganda, Togo, Tanzania, Ivory Coast, Cameroon, China, India, Indonesia, Vietnam	PCA, PLS-DA	Fatty acids, caffeine, acetate, organic acids, trigonelline, chlorogenic acids	[17]
HR-CS-AAS	Roasted	9	Brazil, Ethiopia, Colombia, India, Cuba, Mexico, Honduras, Guatemala, Kenya, Papua NG, Timor, Mussulo, China	ANOVA, CDA	Ca, Mg, Na, K, Fe, Mn	[27]
PTR-TOF-MS	Roasted	6	Brazil, Ethiopia, Guatemala, Costa Rica, Colombia, India	PCA, RF, PDA, dPLS, SVM, ANOVA	H_3_O^+^, NO^+^, O_2_^+^	[28]
NIRS	Green	90	4 different Brazil coffee-producing regions	PLS-DA	Sucrose, lipids, amino acids, caffeine, trigonelline, chlorogenic acids	[29]
UPLC-MS	Green	100	4 different Ethiopia coffee-producing regions	PCA, LDA, ANOVA	Chlorogenic acids	[30]

ICP-AES, Inductively Coupled Plasma Atomic Emission Spectroscopy; EA-P-IRMS, Elemental Analysis-Pyrolysis-Isotope Ratio Mass Spectrometry; NIRS, Near-Infrared Spectroscopy; GC-TOF-MS, Gases Chromatography-Time-of-Flight-Mass Spectrometry; HPLC, High Performance Liquid Chromatography; LC-MS, Liquid Chromatography-Mass Spectrometry; GC-FID, Gas Chromatography-Flame Ionization Detector; MC/ICP/SFMS, Multiple Detector/Collector-Inductively Coupled Plasma-Sector Field Mass Spectrometry; NMR, Nuclear Magnetic Resonance; FT-IR, Fourier Transform Infrared Spectroscopy; PCA, Principal Component Analysis; CDA, Canonical Discriminant Analysis; DFA, Discriminant Function Analysis; ANN, Artificial Neural Network; LDA, Linear Discriminant Analysis; CART, Classification and Regression Tree; PLS-DA, Partial Least Squares-Discriminant Analysis; OPLS-DA, Orthogonal Partial Least Squares-Discriminant Analysis; SIMCA, Soft Independent Modelling of Class Analogy; MLP, Multilayer Perceptron; RF, Random Forest; PDA, Penalized Discriminant Analysis; dPLS, Discriminant Partial Least Squares; SVM, Support Vector Machines.
